# Permutation Entropy and Statistical Complexity in Mild Cognitive Impairment and Alzheimer’s Disease: An Analysis Based on Frequency Bands

**DOI:** 10.3390/e22010116

**Published:** 2020-01-18

**Authors:** Ignacio Echegoyen, David López-Sanz, Johann H. Martínez, Fernando Maestú, Javier M. Buldú

**Affiliations:** 1 Laboratory of Biological Networks, Centre for Biomedical Technology, Universidad Politécnica de Madrid (UPM), 28223 Madrid, Spain; jmbuldu@gmail.com; 2 Complex Systems Group, Rey Juan Carlos University, 28933 Madrid, Spain; 3 Grupo Interdisciplinar de Sistemas Complejos (GISC), 28911 Madrid, Spain; johemart@gmail.com; 4 Laboratory of Cognitive and Computational Neuroscience, Centre for Biomedical Technology, Universidad Politécnica de Madrid (UPM), 28223 Madrid, Spain; david.lopez@ctb.upm.es (D.L.-S.); fernando.maestu@ctb.upm.es (F.M.); 5 Department of Experimental Psychology, Complutense University of Madrid, 28223 Madrid, Spain; 6 Biomedical Engineering Department, Universidad de los Andes, Bogotá 111711, Colombia; 7 Biomedical Research Networking Center in Bioengineering, Biomaterials and Nanomedicine, 28029 Zaragoza, Spain

**Keywords:** statistical complexity, permutation entropy, Alzheimer’s disease, mild cognitive impairment, regions of interest, frequency bands

## Abstract

We present one of the first applications of Permutation Entropy (PE) and Statistical Complexity (SC) (measured as the product of PE and Jensen-Shanon Divergence) on Magnetoencephalography (MEG) recordings of 46 subjects suffering from Mild Cognitive Impairment (MCI), 17 individuals diagnosed with Alzheimer’s Disease (AD) and 48 healthy controls. We studied the differences in PE and SC in broadband signals and their decomposition into frequency bands (δ, θ, α and β), considering two modalities: (i) raw time series obtained from the magnetometers and (ii) a reconstruction into cortical sources or regions of interest (ROIs). We conducted our analyses at three levels: (i) at the group level we compared SC in each frequency band and modality between groups; (ii) at the individual level we compared how the [PE, SC] plane differs in each modality; and (iii) at the local level we explored differences in scalp and cortical space. We recovered classical results that considered only broadband signals and found a nontrivial pattern of alterations in each frequency band, showing that SC does not necessarily decrease in AD or MCI.

## 1. Introduction

Alzheimer’s disease (AD) is one of the most common diseases in Western societies and by far the most prevalent form of dementia, with ∼60% to 80% of all registered cases being AD type [1]. It entails an enormous outlay for the patient’s familiar unit, as well as for healthcare systems and governments. As a neurodegenerative disease, the condition progresses slowly in a continuum, with physiological changes starting decades before the appearance of cognitive symptoms [2]. Early manifestations of AD are related with an increasing difficulty in remembering conversations, proper names or events and are often accompanied with apathy and depression. Later symptoms include temporospatial disorientation, poor decision-making, behaviour and personality changes. In final stages, speaking, walking and even swallowing are almost impeded [1]. Physiologically, the two hallmarks of AD are a progressive accumulation of amyloid-β (Aβ) in neuropil [3,4] in the form of neuritic or diffusive plaques [5] and formation of neurofibrilary tangles of phosphorylated-tau (p-tau), a protein involved in microtubules stability [3,6]. Aβ deposition usually starts in frontal and temporal lobes (especially entorhinal and ventral frontal cortices), while p-tau is more prone to be accumulated in the hippocampal formation, limbic system and entorhinal cortex [3]. The progression of the disease courses with expansion of these processes across the entire cortex, leading to neuronal death and volume loss, with the subsequent disconnection due to synapse destruction [7]. This fact led some authors to refer to AD as a disconnection syndrome [8], where the massive loss of synapses would be the main reason behind memory deficits. Nonetheless, the first stages of AD are characterized by neuronal hyperactivity and hypersynchrony, due to the fact that Aβ plaques act selectively on pyramidal cells [5]. Pyramidal cells are the most common type of cortical neuron and different axonal systems innervate them in different regions. Generally speaking, axon terminals in contact with the perisomatic region of pyramidal cells are GABAergic, with an inhibitory function. This is precisely where Aβ plaques contact neurons. The net effect of this accumulation selectively acting on inhibitory synapses is hyperactivity, because inhibition ceases, which derives in a global disruption of the excitatory/inhibitory balance [9]. This imbalance seems to be reflected in an impairment of ultra slow frequencies (<1 Hz), associated with non-REM sleep and memory consolidation and systematically associated to memory decline in mice models [9] and humans [5]. In more advanced stages of the disease, synchronization and activity decreases [10] and symptomatology worsens.

Given that AD spans for decades, many efforts have been made on detection and treatment of early stages. The one that has received more attention is amnestic mild cognitive impairment (MCI), as it is a clinical condition between normal aging and AD, in which individuals suffer memory loss (greater than expected due to age) but the cognitive function is preserved and the criteria for probable AD is not met [4]. People in this condition are at higher risk of developing AD in comparison to age-matched population [11,12], with a conversion ratio of ∼10%–15% per year and more than 80% after six years [4]. Nevertheless, the underlying mechanisms by which only a portion of them progress to AD (progressive MCI) while others stay in it (stable MCI) are still poorly understood.

Biospecimen studies have focused on p-tau and Aβ concentrations in cerebrospinal fluid (CSF) to predict onset and discern between early-stage AD and non-demented aging [13,14]. Positron emission tomography (PET) analyses have consolidated the idea that Aβ deposition begins decades before dementia, preceding cognitive decline and brain atrophy, which can be used to predict the evolution of the symptoms and the conversion from MCI to AD [2]. Also, many efforts have been devoted to understand how measurable properties and observables of biomedical signals change between groups of demented and non demented elders. For example, in electroencephalography (EEG) and magnetoencephalography (MEG) data, it has been found that frequencies are altered, either peaks [15,16,17,18,19], means within a frequency range [20,21], or medians (were the power spectrum can be divided into two sections of equal total power) [22,23] tend to decrease in AD when compared to controls. The signal’s power is also altered: absolute power within frequency ranges (bands) tends to increase in low frequencies and decrease in high ones [15], especially in delta and theta bands [24,25,26,27]. The same tendency is observed in relative power, in proportion to total power [18,21,28,29,30].

Entropy and information theoretic measures have received some attention too, dealing especially with EEG and MEG data. Many studies pointed to the same direction: entropy and complexity tend to be decreased in AD patients and their signals become more predictable. For example, Gómez et al. [31,32] and Hornero and his collaborators [33] found that AD patients had, in group-average, a diminished auto mutual information (AMI) decreasing rate, with local differences found in many channels. The AMI decreasing rate is an indicator of how predictable future values of a time series are, based on past ones. Also, sample entropy (SamEn) [33,34,35], approximate entropy (ApEn) [33,36], spectral entropy (SpecEn) [19,21,22,23,37,38], Lempel-Ziv complexity (LZC) [23,33,35,39,40,41] and Lopez Ruiz-Mancini-Calbet (LMC) complexity [37] have been found to be decreased in AD patients. All these studies took into account broadband MEG/EEG signals at the scalp level (sensors), finding heterogeneous, local differences, in a few channels or areas. Only in References [36,41,42] were frequency bands taken into account, finding statistical differences in delta, theta and low beta bands, respectively.

In the present paper, we propose the use of statistical complexity (SC), a measure that combines permutation entropy (PE) with the Jensen divergence as a disequilibrium metric, to study how MEG signals differ when comparing AD, MCI and age-matched controls, paying attention not only to broadband time series but to its decomposition in frequency bands, namely delta, theta, alpha and beta and its estimation both at the sensor and the source levels, the latter by inferring the dynamics of the so-called *regions of interest* (ROIs). All details concerning the calculations of SC, PE and signal manipulations can be found in Section 4.2 and Section 4.3. Regarding the use of PE to study dementia, Labate and his colleagues [43] applied it to EEG broadband signals, finding a tendency towards a loss in complexity (AD < MCI < CG). Deng and her colleagues [42] used a weighted version of permutation entropy on 16-channels EEG data from AD and controls, finding the dynamics recorded from the AD group less complex in the theta band. A multivariate multi-scale version of it was proposed in Reference [44], where the authors took into account various channels to get the corresponding ordinal patterns distributions. They concluded that it might be useful to detect “slowing” effects (in terms of frequencies) related to the disease. PE has also been used on fMRI data [45], where the authors explored how the brain entropy maps [46] changed among groups (controls, early MCI, late MCI and AD). It has also been used to properly measure the time reversibility aspects of epilepsy EEG data [47]. Statistical comparisons revealed that AD patients tend to have lower values of PE than MCI and controls. Apparently, these differences are clustered in occipital, frontal and temporal lobes, indicating that the alteration in complexity is not homogeneous and depends on the region under study. On the other hand, the Jensen divergence was used in Reference [48] as a proxy for time series irregularity or disorder. In this work, authors applied the Jensen divergence on a set of MEG recordings from controls, MCI and AD subjects, concluding that AD had higher values of disorder but the alteration was spatially heterogeneous.

To the best of our knowledge, our proposal, along with the one recently proposed in Reference [41], is a novel and more complete approach to understand AD and MCI, given that previous efforts have focused only on broadband signals instead of frequency bands and comparing usually two groups (typically, AD vs. controls or MCI vs. controls). In turn, we compare the results obtained at the sensor and source level, showing the differences (and similarities) between both methodologies.

## 2. Results

Our database is composed of MEG recordings from 17 Alzheimer’s disease patients, 46 Mild Cognitive Impairment subjects and 48 age-matched healthy controls (CG). Each time series is decomposed in four frequency bands (δ: 1–4 Hz; θ: 4–8 Hz; α: 8–13 Hz and β: 13–31 Hz) and its permutation entropy and statistical complexity are calculated. Both metrics (PE and SC) have proven to be robust and fast methods to quantify the organizational properties and temporal structure of dynamical systems [49]. As detailed in Section 4.3, we obtained one value of SC for each area (sensor or ROI) in each subject and performed our analyses at three levels. First, we observed how the parameter under study behaved at the global level, taking into account all values and subjects, and comparing it between frequency bands. Second, we averaged across subjects and compared complexity between groups at each frequency band. And lastly, we observed how differences were locally distributed in the brain.

**Figure 1:**
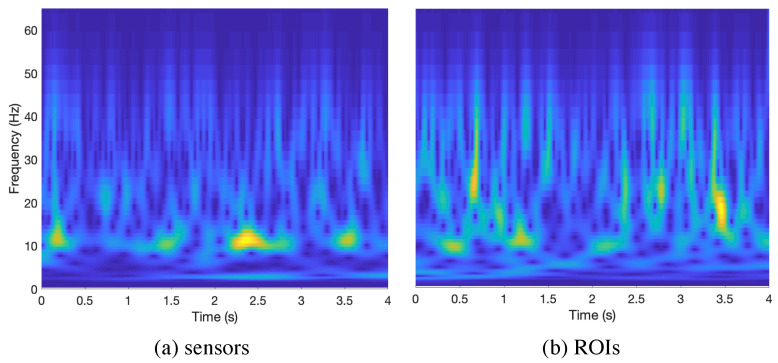
Example of a spectrogram from a signal in sensors (**a**) and regions of interest (ROIs) (**b**). Selected from a randomly chosen channel/ROI of a healthy subject.

Figure 1 shows the spectrograms from a channel (randomly selected) and a sample in the control group, for sensors (left) and ROIs (right). Although there are some unavoidable differences due to the source estimation procedure, most of the frequency components are preserved and kept in time. This example shows how the α rhythm (that peaks at 10 Hz) dominates over any other, something present in all recordings (although we only show one here to provide the reader with some evidence). This fact has important implications in further analyses, as the α rhythm will be the band better characterized, due to its stronger signal-to-noise ratio.

**Figure 2:**
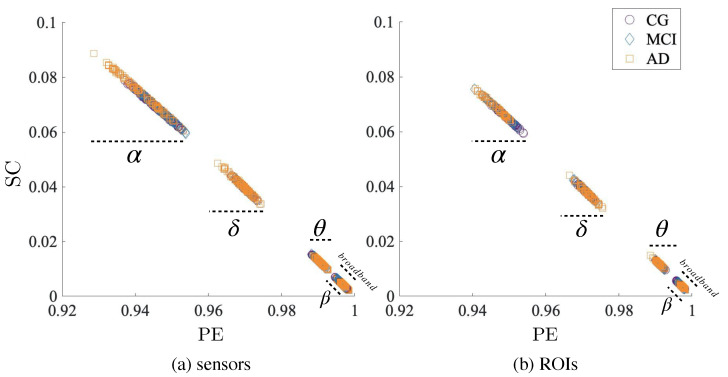
Entropy-complexity [PE,SC] planes for sensors (**a**) and ROIs (**b**), for the three groups (control group, CG; mild cognitive impairment, MCI; and Alzheimer’s disease, AD). Both modalities show the same qualitative results. There are clear differences between bands, especially in the case of α, δ and θ. Each band seems homogeneous across groups and modalities.

In Figure 2, we can observe the entropy-complexity [PE,SC] plane for the broadband signal and each frequency band in each group. Left hand side plot corresponds to sensors, while right hand side is for ROIs. Each color and shape in the figure corresponds to one group (CG; MCI; AD). We can clearly observe a stable pattern across modalities and groups: bands exhibit different levels of complexity, α being the most complex band and β the least complex. Also, the fact that the pattern is qualitatively the same disregarding the modality (sensors or ROIs) points to the conclusion that the method is robust and the signal is reasonably well preserved. Interestingly, when the whole signal is taken into account (broadband), the values of complexity are notably decreased. This fact might indicate that analyzing the time courses without previous decomposition into its frequency components could hide the variability contained in the frequency bands.

**Figure 3:**
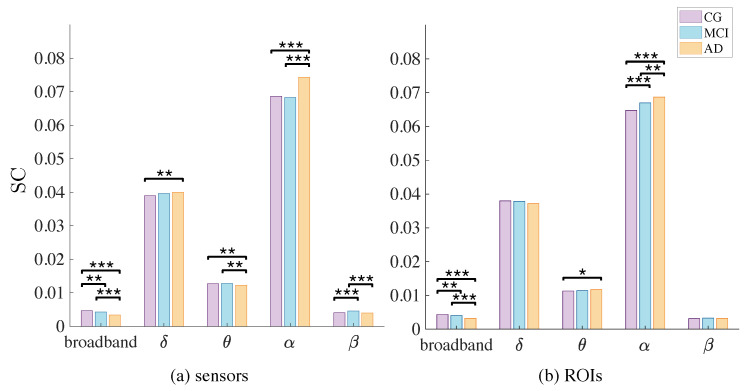
Average complexity (SC) for broadband, δ, θ, α and β bands, for sensors (**a**) and ROIs (**b**). One asterisk: p<0.05, two asterisks: p<0.01, three asterisks: p<0.001. We find differences between the three groups in broadband, in both modalities. Also, θ and α bands show clear differences between CG and AD and MCI and AD, a pattern only preserved in α band in ROIs.

In Figure 3 we show the result of a non parametric one-way ANOVA (Kruskal-Wallis test) for each band. Statistical differences are marked with one asterisk (p<0.05), two (p<0.01) or three (p<0.001). As we can observe, the pattern of differences is, again, very similar between modalities. In sensors, major differences are found in broadband, θ, α and β. Note how each band follows a different pattern in groups’ averages. In broadband and θ, we find a decreasing pattern (the more advanced the disease, the lesser the complexity). Surprisingly, this pattern is almost inverted in α, where the AD group shows higher values of complexity in comparison with the other two. The case of β is also different: MCI shows higher complexity, with CG and AD being indistinguishable from one another. Some of these differences are not present in ROIs: θ and β do not exhibit clear differences; broadband behaves qualitatively equal to sensors and an upwards tendency is observed in α, with AD showing higher complexity than MCI and CG and MCI higher than CG.

Interestingly, broadband shows clear statistical differences, in the same direction as established previously in the literature: complexity (no matter the method) decreases as the disease steadily draws on. Surprisingly, this is not necessarily the case when each frequency band is observed individually. All these results are summarized in Table 1 and Table 2.

**Figure 4:**
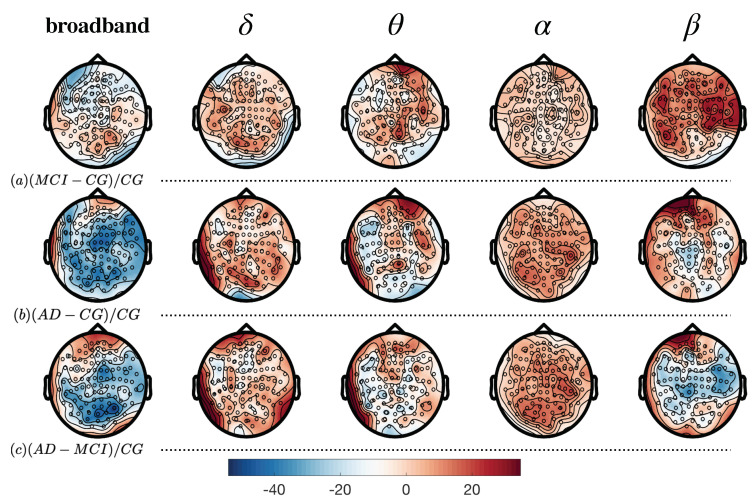
Topographic map of complexity over the scalp for each band and group. Each dot represents a sensor. First row (**a**): values in complexity as the difference between MCI and Controls divided by controls ((MCI−CG)/CG). Second row (**b**): difference between AD and Controls divided by controls ((AD−CG)/CG). Third row (**c**): difference between AD and MCI divided by controls ((AD−MCI)/CG).

**Figure 5:**
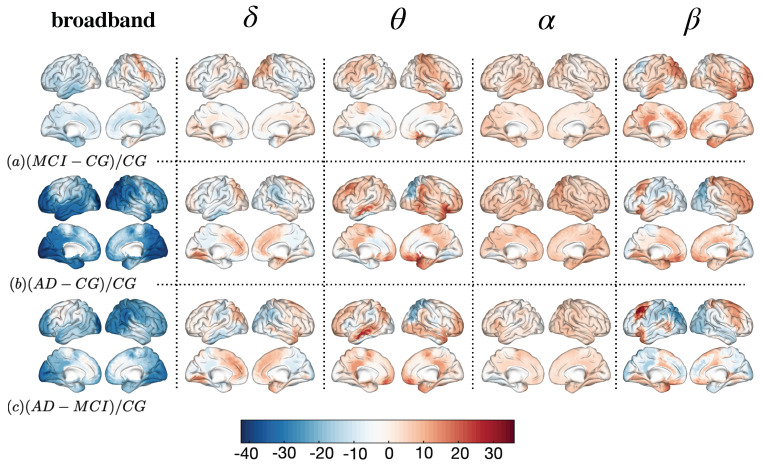
Distribution of complexity over the cortical surface for each band and group (estimated sources). First row (**a**): values in complexity as the difference between MCI and Controls divided by controls ((MCI−CG)/CG). Second row (**b**): difference between AD and Controls divided by controls ((AD−CG)/CG). Third row (**c**): difference between AD and MCI divided by controls ((AD−MCI)/CG).

Figure 4 and Figure 5 show local differences at the scalp level (sensors) and in cortical areas (ROIs), respectively. We can only obtain one value of complexity per site (sensor or ROI) and subject (see Section 4.3 for more details). Hypothesis testing with such fewer data is not recommended and we prefer to safely explore differences qualitatively. A visual inspection is interesting to understand spatial tendencies of the complexity parameter. Both figures reflect the percentage of change at each region, normalized with the control group. Each site (sensor or ROI) is the difference in complexity between the groups to be compared, normalized by the control group and rescaled to be between 0 and 100 (as a percentage of variation). First row (*a*) depicts the differences between MCI and controls ((MCI−CG)/CG×100), second row (*b*) shows differences between AD and controls ((AD−CG)/CG×100) and third row (*c*) differences between AD and MCI ((AD−MCI)/CG×100). Each column shows the differences at each band or signal considered: *broadband*, δ, θ, α and β.

Figure 4 shows the topographic distribution of complexity over the scalp. Note how each column has its own unique pattern of differences, not necessarily homogeneous across groups. In broadband, the AD group has smaller values than controls and MCI, especially in frontal and parietal sensors, around the sensoriomotor ones (see broadband, rows *b* and *c*), with variations up to 40%. On the contrary, most rostral section of frontal and left temporal sensors show higher values of complexity in AD than in controls or MCI (especially comparing with the latter). It is interesting to note how these differences are not present between MCI and controls (*a*), indicating that both groups are more homogeneous in broadband. δ band also presents more homogeneity between MCI and controls, while AD has higher values of complexity in comparison to the other two groups in left temporal and occipital sensors. Also, in the most anterior area of the frontal sensors, AD shows higher values of complexity than MCI. θ band also shows a left temporal pattern of higher complexity in AD against both controls and MCI, while frontal sensors seem to be more complex when considering MCI and AD against controls but not AD against MCI. Surprisingly α seems to show a more homogeneous pattern, all over the scalp, with differences around 10% of variation but with no clear spatial differences. Lastly, β band seems to show more complexity in MCI stages than in AD. In the first row, the MCI group shows clearly more complexity than controls, especially in parietal and right temporal sensors. AD presents more complexity in left frontal sensors in comparison to controls and MCI but in general is less complex than MCI in parietal ones. Main findings regarding these comparisons can be found in Table 3.

In Figure 5 we show the spatial distribution of complexity in the source reconstruction, corresponding to the time series estimated in ROIs. At first sight, differences are somewhat smoother, although preserved with respect to Figure 4. In general, MCI is less complex than controls (around 10%) in broadband, except at the right premotor area. Also, controls have more complexity in comparison to AD (*b*), especially in prefrontal and occipital areas: visual (I, II and III) and association cortex (temporo-and parieto-cingular pathways). This tendency is also observable between AD and MCI (third row) but reverted in the other bands: δ appears to be more complex in MCI, especially in occipito-parietal cortex. On the other hand, prefrontal, premotor and occipital areas show more complexity in AD in comparison to the other two groups. In θ we can observe a tendency in the premotor area and the frontal inferior gyrus. The latter is present when comparing MCI and CG but especially prominent between AD and CG, indicating that the complexity in that area increases with as the disease advances. It is also notable how the ventral anterior cingular cortex (subgenual section) is more complex in AD than in CG and also slightly more complex in AD than in MCI. On the contrary, the secondary somatosensory cortex and the occipito-parietal gyrus are less complex in AD, disregarding the group to be compared with. Again, α band shows a much more homogeneous patterns, where MCI and AD are more complex than controls and AD slightly more than MCI (between 5% and 10% more). On the other hand, β band shows a completely different pattern, with MCI being more complex than AD and CG. As shown in the first row, prefrontal and occipital areas, along with the frontal inferior gyrus show more complexity (MCI vs. CG). AD presents more complexity in prefrontal and anterior cingulate areas, whilst occipital cortex is more complex in CG. In the third row we can observe how occipital areas are less complex in AD than in MCI, while again in the premotor area (especially in the left hemisphere) and in the frontal inferior gyrus this pattern is inverted. All these results are summarized in Table 4.

## 3. Discussion

In this work, we presented an application of a statistical complexity measure to MEG registers from AD and MCI patients, compared to control subjects. First, we showed how each frequency band depicts its own entropy-complexity profile, which is robust against modalities and it is qualitatively the same in the three groups. This fact might indicate that each rhythm has its own dynamical patterns, inherent to the rhythm itself and not necessarily to the modality of the data and the stage of the disease. Nevertheless, it should be noted that the power and spatial distribution of each band will depend upon other physical conditions, such as wakefulness state and the specific task being conducted. Hence, our conclusions must be taken with caution and secluded to resting state. The idea that frequency bands may have different inherent values of complexity that, nonetheless, depend upon the task being carried out is in line with previous studies analyzing task-dependent EEG recordings [50,51]. Indeed, in Reference [51], the authors found that the whole signal, $\beta_{2}$ and α behave in the same qualitative order as they do in our causal planes, even though they analyze task-related data from EEG, using a different metric for the disequilibrium.

The finding that broadband signals show differences between groups is not surprising, as previous studies focused on whole signal analyses agree reporting that complexity tends to decrease as the disease progresses, regardless of the modality of the data (EEG/MEG) and the measure of complexity. For a comprehensive review, see References [52,53]. Also, the fact that ROIs and sensors show the same differences points out the homogeneity of the data and the robustness of the method.

In spite of it, the decomposition in frequency bands does indeed introduce some important changes, especially in θ, α and β. As shown in Figure 3, θ band appears to be different between groups only in sensors, while these differences are lost in ROIs. Interestingly, in this band we cannot see differences between MCI and CG, while MCI versus AD and CG versus AD seem to be different (having AD less complexity). θ oscillations (together with γ rhythms) have been associated to encoding and retrieval of episodic memories, allowing cortical-hippocampal top-down control [54]. Along with the α rhythm, θ has been found to be involved in long range fronto-parietal connections during working memory and mental imagery (again, top-down processing), especially in medial prefrontal regions [55], where Aβ and p-tau tend to deposit first and more abundantly [6]. Although globally (in average) we can state that AD is less complex in this band in comparison to MCI and controls (Figure 3), when observed locally (Figure 4 and Figure 5), we can appreciate that precisely in those areas the pattern is the opposite. We would need more data to conduct a proper statistical analysis at this level, but nevertheless it is worth noting this alteration. Areas consistently reported as the first to be populated with neurofibrillary tangles and neuritic plaques tend to be more complex in the θ band, which has been associated to memory load, maintenance and recovery. Whether or not this is a compensatory mechanism or a direct product of Aβ and p-tau deposition and volume loss is a matter of debate. To the best of our knowledge, this is the first time such a result is reported and definitively needs further analysis.

The case of α is especially hard to interpret, as it seems to be different in each modality, although in both appears to be significantly different between groups. It is clear that in both cases, the AD group shows higher complexity, with MCI being indistinguishable from controls in sensors and significantly higher in ROIs (that is, in-between controls and AD). This could be because the source estimation procedure is introducing some bias, or because it “cleans” information in sensors, unveiling a real pattern. Comparing different source estimation procedures could give us some insights on this question, although it is out of the scope of the present work. In any case, it is worth noting how, in spite of giving such differences, the topology across the cortex is by far the smoothest of all bands considered, with only central and occipito-parietal regions standing slightly out. Tentatively, this could be due to its functional role and anatomical distribution. α oscillations are present in all cortical structures, as well as in hippocampus and thalamus and have been associated to inhibition and attention processes comprising all the cortex in long-range synchronization patterns [56]. That is, in comparison to more local oscillations, such as γ or cortico-hippocampal θ rhythms, α band is usually defined as a more global, long-range phenomenon.

β band, on the other hand, seems to be different between groups only in sensors, but not in ROIs. Again, more source estimation methods could give us a better idea of the extent to which such differences come from real different dynamics, or simply due to the procedure. In this case, local differences tend to be in frontal areas, near sensoriomotor cortex. Results in this band are not aligned with those presented in Reference [41] using the Lempel-Ziv entropy. With similar groups (controls (CN), MCI and subjective cognitive decline (SCD)) recruited in the same facilities, a similar preprocessing pipeline, source reconstruction technique and anatomical atlas (Harvard-Oxford), they found that complexity was decreased in the low beta band (12–20 Hz) in two comparisons: CN vs. MCI, especially in cingulate, precuneus and superior parietal lobe; and SCD vs. MCI, with differences in precuneus and posterior cingulate. On the contrary, we only found differences in sensors (ROIs were not significant), with MCI showing more complexity than the other two groups. However, a direct comparison between both results is not recommended, due to the fact that sensors are distributed over the scalp and do not correspond unlikely to cortical sources. Furthermore, it is worth noting that the sources estimated in Reference [41] are not exactly the same, the measure to quantify complexity is different (they use LZC), groups are not the same either and the subdivision of beta is something we did not apply. Therefore, the differences found might be a consequence of the methodological procedure. While there could be real differences in low beta, it is likely that we could not find them, as we analyzed a broader band altogether.

Finally, we must mention that the results (see, for example, Figure 2) might be affected by the quality of the signal. Clearer differences were found in the α band, which probably has to do with the fact that it is the most predominant frequency component. This results in a higher SNR, which in turn affects our ability to find differences between groups. The opposite also holds true: a lack in power in other frequency bands might hinder our capability of finding differences. On the other hand, having only 17 subjects in the AD groups prevents us from conducting robust statistical analyses at the local level, which definitely limits our ability to get new insights into the matter. Also, having only one group of MCI (instead of two or more follow-up sessions) alters the robustness of our conclusions and does not allow us to quantify how complexity changes over time. For such conclusions, longitudinal datasets should be analyzed in further studies.

## 4. Materials and Methods

### 4.1. Recruitment

A total of 111 volunteer elder adults enrolled this study. All of them were recruited from three different centers, the Center for Prevention of Cognitive Impairment, the Seniors Center of Chamartín District and Hospital Clínico San Carlos Neurology Department in Madrid (Spain). After a complete explanation of the details, aims and protocols that would be followed during the project, all the participants signed an informed consent. All the procedures employed in this research were performed in accordance with approved guidelines and regulations and the Hospital Universitario San Carlos Ethic Committee approved the study.

Participants were divided into three different groups accordingly to their cognitive status; 48 of them were classified as healthy controls (control group, CG) without any sign of cognitive impairment, 46 met criteria to be classified as having amnestic mild cognitive impairment (MCI) while the remaining 17 received a diagnosis of dementia due to Alzheimer’s Disease (AD).

Overall health status was assessed according to a set of tests including the Mini Mental State Examination (MMSE), the short form of the Geriatric Depression Scale (GDS), the Functional Assessment Questionnaire (FAQ) and the Hachinski Ischemic Score. All participants underwent a thorough neuropsychological assessment to evaluate their performance in the different cognitive domains. As a result of this assessment, MCI patients were classified according to the criteria proposed by Petersen [57] and Grundman [58] and all of them presented a significant impairment in the memory domain, thus being considered as amnestic MCI, a subtype known to be at higher risk of AD [59]. All patients classified as AD fulfilled NINCDS-ADRDA criteria of probable AD.

Clinicians ensured that none of the participants met any of the exclusion criteria listed to rule out possible confounders of cognitive decline other than dementia due to AD or other common conditions that could lead to synaptic disruption. More concretely, the exclusion criteria were: (1) history of psychiatric or neurological conditions, or drug consumption that could interfere with normal MEG activity; (2) a modified Hachinski Ischemic Score equal or higher to 5; (3) presence of infection, focal lesions or infarction as evidenced by a neurologist in the individual T2-weighted MR image and (4) history of alcoholism, chronic use of neuroleptics, narcotics, anticonvulsants, anxiolytics or sedative hypnotics.

### 4.2. Data Acquisition and Preprocessing

Each subject underwent a recording of four minutes of resting state brain activity while sitting comfortably with their eyes closed. Magnetic brain signals were acquired using 306 channel Vectorview MEG System with 102 magnetometers and 204 gradiometers (Elekta AB, Stockholm, Sweden) at the Laboratory of Cognitive and Computational Neuroscience in Madrid. The MEG system is placed inside a VacummSchnelze GmbH two layer shielded-room so that external sources of magnetic noise are minimized reducing their contribution to the acquired signals.

Before MEG recording, a brief setup for each subject included the placement of two electrooculogram electrodes (EOG) above and below the eye to capture blinks and eye movements. Additionally, four head position indication (HPI) coils were located on the head surface to estimate head position during the recording. With this aim, the head shape of each participant and the position of three reference points (nasion, left preauricular and right preauricular) together with the exact location of the four HPI coils was localized using a three-dimensional Fastrak digitizer (Polhemus, Colchester, Vermont).

Sampling rate during MEG signal recording was set to 1000 Hz using an online anti-alias filter between 0.1 and 330 Hz. After MEG recording, an offline filtering was applied (tempo-spatial filtering algorithm; tSSS, correlation window 0.9, time window 10 s) [60,61] to subtract the sources of noise placed outside the head. Head movements were corrected using the same algorithm.

Due to tSSS filtering procedure signals recorded by magnetometers and gradiometers are highly correlated and redundant [62], thus only data coming from the 102 magnetometers were employed for further analyses. An automatic algorithm from the Fieldtrip package was used to detect noisy samples containing ocular, muscular or jump artifacts [63] which were visually confirmed by a MEG expert in a second step. Furthermore, an ICA-based procedure was employed to remove ocular and electrocardiographic contributions to the MEG signal. Finally, clean data were segmented into 4 s epochs. To ensure that the number of trials did not affect the results, only subjects with at least 30 clean epochs were included in the analysis. For those subjects with more data available, 30 trials were randomly selected to match the number of epochs used for each group. Hence, trials are not necessarily consecutive in time. Further analyses revealed that in three subjects, one trial was too noisy to be considered, so one random trial was excluded from the analyses in all subjects. Thus, the final number of trials for all subjects was 29.

A T1-weighted MRI was acquired for each participant in a General Electric 1.5 Tesla magnetic resonance scanner. This system uses a high-resolution antenna and a homogenization PURE filter (Fast Spoiled Gradient Echo sequence, TR/TE/TI = 11.2/4.2/450 ms; flip angle 12${}^{\circ }$; 1 mm slice thickness, 256 × 256 matrix and FOV 25 cm).

Clean epochs for each subject were band-pass filtered between 1 and 45 Hz so that low frequency and power line artifact contributions were discarded. Each segment included 4000 samples of real signal (4 s) at each side to prevent edge artifact inside the data due to band-pass filtering.

A realistic model using single-shell was employed to generate the forward model of a 1-cm spacing grid with 2459 sources inside the brain. Magnetic activity at the brain level was reconstructed in each of the nodes by using Linearly Constrained Minimum Variance (LCMV) beamformer [64]. Once time series for all of the 2459 sources were calculated, a representative time-course for each cortical region of a reduced version of the Harvard-Oxford atlas [65] was chosen by selecting the centroid source of each region. As a result, 62 time series (i.e., the number of cortical ROIs included in the analyses) at each trial were used for further analyses in the source space.

For all subjects in both modalities, we conducted a frequency decomposition of all signals, to get an estimate of each time series into four classical frequency bands: δ: 1–4 Hz; θ: 4–8 Hz; α: 8–13 Hz and β: 13–31 Hz. In order to estimate the signal for each frequency band we simply computed the Fourier transform of the signal and reconstructed it via inverse Fourier transform in the range of frequencies specified above. γ band was disregarded for all subjects due to lack of power in that frequency range (31–50 Hz).

**Figure 6:**
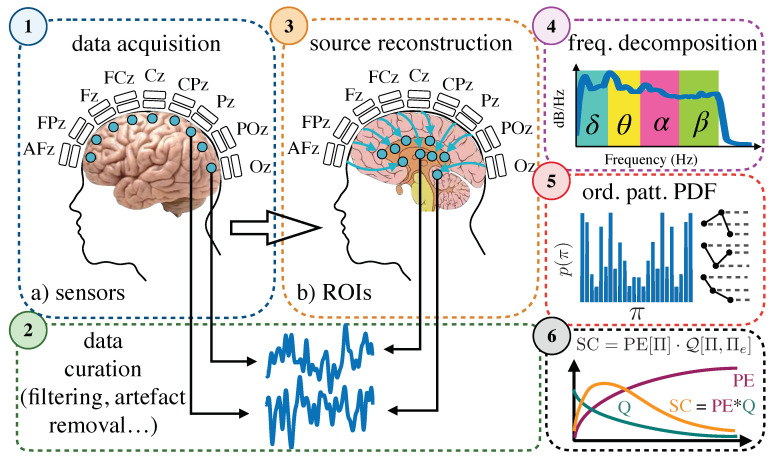
Schematic representation of the pipeline followed to conduct this research. (**1**) Data acquisition from MEG recordings (sensors). (**2**) Data curation. (**3**) Estimated signals in Regions of Interest. (**4**) Frequency decomposition into four different frequency bands: δ, θ, α and β. (**5**) Ordinal patterns extraction and probability density estimation. (**6**) Entropy, disequilibrium and complexity calculation.

To sum up, for each of the 111 subjects (48 CG, 46 MCI and 17 AD), we had 29 trials with time series of 4 s long each (4000 points at a sampling frequency of 1000 Hz) in two modalities: (1) 102 sensors, at the scalp level or its reconstructed signal, (2) 62 Regions of Interest (ROIs). Each signal was decomposed into four frequency bands (δ; θ; α and β) and analyzed separately. A schematic representation of the process can be found in Figure 6.

### 4.3. Permutation Entropy and Statistical Complexity

The goal in the present work is to compare the dynamics of three groups of individuals: controls, mild cognitive impairment and Alzheimer’s disease. From the wide spectrum of options at disposal, we selected the permutation entropy (PE) and statistical complexity (SC), given their simplicity, robustness and feasibility in computation [49]. They have been widely used in diverse fields as econophysics [66,67,68] and biomedical signals [50,51] and have proven to be a very useful tool to unveil subtle differences between stochastic and chaotic dynamics [69]. The first version of the SC was proposed by López-Ruiz, Mancini and Calbet [70]. The authors designed a function to quantify the classical sense of complexity in a statistical fashion, that peaks only when the system is halfway between a perfectly ordered state (i.e., a perfect crystal) and an absolutely random one (as in a perfect gas). In both extremes (perfectly ordered or completely random) the function must be zero. Their contribution was to conceptualize this notion in terms of information and distance to the equiprobable distribution. A perfectly ordered system will contain minimal uncertainty, as very little information is needed to characterize it completely; also, there will only be one preferred state over any other, thus the system will remain in it. On the other hand, a completely disordered system in which every state is equally probable will require maximal information to be described (hence the uncertainty is maximal). The authors’ choice to calculate complexity, based on these two notions are the classical Shannon entropy and a quasi-metric of disequilibrium, a quadratic distance between the systems’ distribution and a corresponding equiprobable distribution of the same size.

The original proposal uses Shannon entropy as a measure of uncertainty, inversely related to the amount of information needed to predict the system’s behaviour. This magnitude is obtained from the probability distribution function (PDF) of any observable in the system, usually notated as $S\left[P\right]=-\sum_{i=1}^{M}p_{i}\ln\left(p_{i}\right)$, where *M* refers to the degrees of freedom of the system. This magnitude has been used in many contexts, and it is still of great utility. Yet, it has three drawbacks [49]: (i) it does not account for any temporal structure present in the data, although important information might be codified in the temporal dynamics; (ii) the PDF must be completely specified beforehand; and (iii) it has proven to work fine only with linear systems, failing to describe chaotic regimes.

These reasons led Bandt and Pompe [71] to develop the *permutation entropy* (PE) measure. It is simple, robust, codifies temporal relationships in the time series and requires no prior knowledge of the underlying distribution. To obtain it, neighbouring values in the time series are compared, generating a symbolic sequence that codifies these cardinal relations. The PDF is calculated over these symbolic sequences, incorporating temporal patterns in it (encoded in the vicinity comparisons). More specifically, at each time *s* of any time series $X=x_{t}:t=1,\ldots ,N$, a vector containing the *D*-th adjacent values is obtained:

(1) $$s\mapsto (x_{s},x_{s+1},\ldots ,x_{s+(D-2)},x_{s+D-1})$$

*D* is the *embedding dimension*, related to the amount of information contained in each vector. It is possible to take non-subsequent values, introducing a time delay τ, related to the autocorrelation function and the intrinsic time delay of the system [72]. For the purposes of our research, we will focus on τ=1 (that is, with no delay, taking into account only subsequent values from the time series).

Each vector is converted into an ordinal pattern, a permutation of the order of D−1, $\pi =(r_{0}r_{1}\ldots r_{D-1})$, that corresponds to the original values sorted in ascending order:

(2) $$x_{s+r_{0}}\leq x_{s+r_{1}}\leq \ldots \leq x_{s+r_{D-2}}\leq x_{s+r_{D-1}}$$

As an example, take the time series X={4,5,1,6,5,1,9}. For D=3, at s=1 we will have the vector (4,5,1), that in ascending order would be (1,4,5). The ordinal pattern corresponding to it would be the permutation π=(1,2,0). When two values are equal, they are sorted in order of appearance. We will work only with non overlapping vectors (consecutive, not sliding windows). Thus, our next vector would be at s=2+(D−1)=4, reading (6,5,1), ordered as (1,5,6), whose ordinal pattern corresponds to the permutation π=(2,1,0). We repeat this procedure until we have converted the whole time series into its symbolic sequences. The number of patterns, as we take only consecutive windows, will be M/D.

There might be *forbidden patterns* (sequences not present), due to the intrinsic dynamics of the system or given by the procedure [49], ruled by the interplay of *D*, τ, intrinsic frequency of the signal, its length and the choice of consecutive or sliding window. The former is interesting, as the study of the presence or absence of each pattern can unveil relevant information about the underlying dynamics of the system. The latter, on the contrary, is a bias in the density estimation and must be avoided, as it will not reflect the real dynamic variability. In general, we can calculate a probability distribution Π encoding the frequency $\pi_{i}$ of each ordinal pattern *i* extracted from the observed time series and study its *Permutation Entropy* (PE):

(3) $$\mathrm{PE}=-\sum_{i=1}^{D!}\pi_{i}\ln\pi_{i}$$

that can be normalized as:

(4) $${\mathrm{PE}}_{norm}=-\frac{1}{\log_{2}D!}\sum_{i=1}^{D!}\pi_{i}\log_{2}\pi_{i}$$

changing the units to bits and given that $PE\in [0,log_{2}D!]$. As stated previously, the number of patterns, when only consecutive windows are considered, will be M/D. The number of patterns will have great impact on the calculation of the PDF and its permutation entropy, and a minimum number of them must be ensured to have robust statistics. As a rule of thumb, Bandt and Pompe [71] propose that D!<<M to estimate entropy reliably.

The other element in the recipe to get the statistical complexity is the disequilibrium (usually denoted as Q), that measures the difference D between the empirical distribution’s entropy and its equiprobable counterpart. Again, there is a panoply of options at disposal to measure this distance. We selected the *Jensen-Shannon divergence*$J[\Pi ,\Pi_{e}]$ [49,73,74] for two reasons. The first one is that the original proposal [70], which calculated D as a simple quadratic distance, has many drawbacks [75,76]. Among others, it does not take into account that the distance is given in terms of statistical space, it is not extensive (it does not grow with the size of the system, thus it does not tend to a constant value in the thermodynamic limit), it cannot distinguish between diverging periodic configurations and more generally, it is insensible to the statistical structure of any regular Markov chain (and any system under this class). These drawbacks led us to the second reason for selecting the Jensen-Shannon divergence: As its square root, it satisfies the triangle inequality [73,77] and, given that it is defined in terms of entropies, it is an extensive quantity in the thermodynamical sense. The Jensen-Shannon divergence is a symmetric version of the Kullback-Leibler entropy and can be written as:

(5) $$\begin{array}{cc} D_{J}\left[\Pi ,\Pi_{e}\right] & =J_{S}\left[\Pi ,\Pi_{e}\right]=\left\{K\left[\Pi |\Pi_{e}\right]+K\left[\Pi_{e}|\Pi \right]\right\}/2\\ \; & =S\left[\frac{\Pi +\Pi_{e}}{2}\right]-S\left[\Pi \right]/2-S\left[\Pi_{e}\right]/2 \end{array}$$

In our case, we will consider the probabilities of the ordinal patterns extracted from the time series under study Π, and its equiprobable counterpart $\Pi_{e}$, where each permutation $\pi_{i}$ has the same probability of occurrence 1/D!.

Finally, the *statistical complexity* (SC) measure that we are using is simply the product of these two quantities:

(6) $$\mathrm{SC}\left[\Pi \right]=\mathrm{PE}\left[\Pi \right]\cdot Q_{J}\left[\Pi ,\Pi_{e}\right]$$

Given the noisy nature of our data (even after being curated, the signal-to-noise-ratio is always less than ideal and it depends on the power of the frequency component under study), obtaining the ordinal patterns directly from the time series is not an option. D=3 or D=4, which are the maximal embedding dimensions considered that still meets the criteria of D!<<M, always induces bimodal probability distributions, with patterns (0,1,2) and (2,1,0) being extremely likely to occur and any other pattern extremely unlikely. Those two patterns correspond to perfectly ordered upward and downward vectors, respectively. This is due to the fact that at that scale (D=3 or 4), the vectors are driven by the inner fluctuations of the signal, present only at local levels (while the envelope of the series is preserved). Such distributions are artificial by construction and do not reflect the natural variability of empirical systems. The later estimation of entropy and disequilibrium will be biased, and any conclusion derived from it, untrustworthy.

To correct this issue, we obtained the ordinal patterns not directly from the time series, but from their local maxima. The number of maxima will depend on the frequency, with slower frequencies having less peaks than faster ones. This procedure ensures a reliable extraction of the ordinal patterns, but reduces the number of points considered notably: From 4000 points in the original time series to 111.74(±9.12) and 121.46(±5.98) in broadband (sensors and ROIs, respectively), 13.58(±0.97) and 13.53(±0.97) in δ band (sensors and ROIs), 26.89(±1.56) and 26.58(±1.55) in θ band, 40.54(±1.58) and 40.68(±1.57) in α band and 88.23(±3.96) and 90.54(±3.5) in β band. This means that in the worst case scenario (∼13 peaks in δ, we can obtain only 4 vectors). To overcome this issue, we gathered the ordinal patterns from all epochs to build the distribution (bear in mind that we have 29 clean epochs for each subject). Thus, again in the worst case scenario, we had 4×29=116 vectors of length D=3 with 3!=6 possible permutations. Building the distributions from all epochs solved the problem of the estimation, but posed another one. We only had one value of complexity per site (sensor/ROI) and subject, losing in the estimation the option of having multiple values of complexity for each channel and subject. Hence, statistical comparisons at this level (site level) were not possible.

### 4.4. Pipeline

As depicted in Figure 6, the pipeline to analyze our data was the following: (i) we acquired the data from the magnetometers, at the scalp level (sensors) and (ii) curated it, which implied spatio-temporal filtering to correct for external noise and head movement, artefact removal, epoching into clean segments of 4 s and band-pass filtering between 1 and 45 Hz. Then, time series (at the sensors level) were (iii) projected into a source space, grouped to fit the Harvard-Oxford atlas and averaged to reconstruct the data at the source level (Regions of Interest). All data, either coming from sensors or ROIs was then (iv) decomposed into four frequency components, namely δ (1–4 Hz), θ (4–8 Hz), α (8–13 Hz) and β (13–31 Hz). From each signal we (v) extracted its ordinal patterns and obtained the corresponding distribution at the epoch-level (constructing the PDF from the ordinal patterns obtained in all trials), thus having one distribution of ordinal patterns per site (sensor or ROIs) and subject at each frequency band. Finally, we (vi) estimated the permutation entropy and disequilibrium associated to the distribution and obtained the statistical complexity measure.

## 5. Conclusions

Complexity in Alzheimer-type dementia and its preclinical stages yielded non-trivial differences, that were heterogeneous over cortical (scalp) areas and estimated sources (ROIs) and strongly depended on the phase of the disease and on the band considered. Here we recovered the same differences reported in other studies for broadband (whole) signals, where complexity decreases with the stage of the condition. Nevertheless, this pattern changed at each frequency band and even when group averages might point in this direction, local differences indicated that complexity can even be higher in AD, especially in areas where deposition of Aβ and p-tau are more prominent. These results, novel in the literature, still need a physiology-based explanation under the light of the physical alterations undergoing the symptoms.

Further studies should focus on collecting more data, especially with longitudinal, follow-up sessions, that enable to carry out robust statistics at the local level and quantify changes in entropy and complexity as a function of time. It would also be of great interest to correlate the observed changes with Aβ levels in CSF samples or hippocampal volume from MRI scans. To give plausible explanations of the increase in complexity in AD, more analyses about entropy and complexity from intra-cortical electrodes in impaired brains should be carried out. More local registers (at meso or even micro-scale) would be a great tool to asses how dynamics change in the presence of Alzheimer’s disease. In view of all, more research is needed to fully understand how the progression of Alzheimer’s disease alters brain dynamics, both spatially and at different frequency bands.

## Figures and Tables

**Table 1:** Summary of groups average complexity (SC) for broadband, δ, θ, α and β bands for sensors. One asterisk: p<0.05, two asterisks: p<0.01, three asterisks: p<0.001. We find differences between the three groups in broadband and between CG vs. AD and MCI vs. AD in θ and α bands.

	Broadband	δ	θ	α	β
CG	0.0046 (*** CG vs. AD) (** CG vs. MCI)	0.0389 (** CG vs. AD)	0.0128 (** CG vs. AD)	0.0687 (*** CG vs. AD)	0.0040 (*** CG vs. MCI)
MCI	0.0043 (** MCI vs. AD)	0.0396	0.0128 (** MCI vs. AD)	0.0683 (*** MCI vs. AD)	0.0046 (*** MCI vs. AD)
AD	0.0034	0.0400	0.0123	0.0743	0.0040

**Table 2:** Summary of groups average complexity (SC) for broadband, δ, θ, α and β bands for ROIs. One asterisk: p<0.05, two asterisks: p<0.01, three asterisks: p<0.001. We find differences between the three groups in broadband and α band.

	Broadband	δ	θ	α	β
CG	0.0043 (*** CG vs. AD) (** CG vs. MCI)	0.0380 (* CG vs. AD)	0.0113	0.0648 (*** CG vs. AD) (** CG vs. MCI)	0.0032
MCI	0.0040 (*** MCI vs. AD)	0.0378	0.0114	0.0670 (** MCI vs. AD)	0.0033
AD	0.0032	0.0372	0.0117	0.0687	0.0032

**Table 3:** Summary of results in topographic map comparisons over the scalp (sensors). First column: values in complexity as the difference between MCI and Controls divided by controls ((MCI−CG)/CG). Second column: difference between AD and Controls divided by controls ((AD−CG)/CG). Third column: difference between AD and MCI divided by controls ((AD−MCI)/CG).

	(MCI − CG)/CG	(AD − CG)/CG	(AD − MCI)/CG
broadband	MCI ≈ CG	AD < CG (frontal; parietal) AD > CG (frontal; left temporal)	AD < MCI (frontal; parietal) AD > MCI (frontal; left temporal)
δ	MCI ≈ CG	AD > CG (left temporal; occipital)	AD > MCI (frontal; left temporal; occipital)
θ	MCI > CG (frontal)	AD > CG (frontal; left temporal)	AD > MCI (left temporal)
α	MCI ≈ CG	AD ≈ CG	AD ≈ MCI
β	MCI < CG (parietal; right temporal)	AD > CG (left frontal)	AD < MCI (parietal) AD > CG (left frontal)

**Table 4:** Summary of results in estimated sources comparisons (ROIs). First column: values in complexity as the difference between MCI and Controls divided by controls ((MCI−CG)/CG). Second column: difference between AD and Controls divided by controls ((AD−CG)/CG). Third column: difference between AD and MCI divided by controls ((AD−MCI)/CG).

	(MCI − CG)/CG	(AD − CG)/CG	(AD − MCI)/CG
broadband	MCI < CG MCI > CG (right premotor)	AD < CG (prefrontal; occipital; association cortex)	AD < MCI (prefrontal; occipital; association cortex)
δ	MCI ≈ CG	AD > CG (occipital; premotor; prefrontal)	AD > MCI (occipital; premotor; prefrontal)
θ	MCI > CG (premotor; frontal inf. gyrus)	AD > CG (premotor; frontal inf. gyrus; ventral ant. cing. cortex) AD < CG (somatosensory ctx.; occipito-parietal gyrus)	AD > MCI (ventral ant. cing. cortex) AD < MCI (somatosensory ctx.; occipito-parietal gyrus)
α	MCI > CG	AD > CG	
β	MCI > CG (prefrontal; occipital; front. inf. gyrus)	AD > CG (prefrontal; anterior cingulate) AD < CG (occipital)	AD < MCI (occipital) AD > MCI (left premotor; frontal inf. gyrus)
